# Approach of Dental Implants Through the  Transfer-Matrix Method

**DOI:** 10.3390/bioengineering13060706

**Published:** 2026-06-19

**Authors:** Rǎzvan Alexandru Mitrea, Mihai-Sorin Tripa, Alexandru Vlad, Iulia-Maria Bărăian, Petre-Corneliu Opriţoiu, Roxana Carmen Cordoş, Carmen-Gabriela Băcilă, Daniela-Corina Jucan, Mihaela Ligia Ungureşan, Liviu Bolunduţ, Dan Pop, Ioana Monica Duncea, Mariana Florica Pop, Honoriu Vălean, Ioan-Aurel Cherecheş, Veronica Mîndrescu, Viorica-Mihaela Suciu, Doina-Iulia Rotaru

**Affiliations:** 1 Department of Mechanical Engineering, Technical University of Cluj-Napoca, 400114 Cluj-Napoca, Romania; razvan.mitrea@rezi.utcluj.ro (R.A.M.); alexandru.vlad@rezi.utcluj.ro (A.V.); 2 Department of Design Engineering and Robotics, Technical University of Cluj-Napoca, 400114 Cluj-Napoca, Romania; mihai.tripa@muri.utcluj.ro; 3 Department of Automation, Technical University of Cluj-Napoca, 400114 Cluj-Napoca, Romania; iulia.baraian@campus.utcluj.ro (I.-M.B.); honoriu.valean@aut.utcluj.ro (H.V.); 4 Department of Land Measurements and Cadaster, Technical University of Cluj-Napoca, 400114 Cluj-Napoca, Romania; petre.opritoiu@mtc.utcluj.ro; 5 Department of Management and Economic Engineering, Technical University of Cluj-Napoca, 400012 Cluj-Napoca, Romania; roxana.cordos@mis.utcluj.ro (R.C.C.); gabriela.bacila@mis.utcluj.ro (C.-G.B.); daniela.jucan@mis.utcluj.ro (D.-C.J.); 6 Department of Physics and Chemistry, Technical University of Cluj-Napoca, 400114 Cluj-Napoca, Romania; mihaela.unguresan@chem.utcluj.ro (M.L.U.); liviu.bolundut@chem.utcluj.ro (L.B.); 7 Department of Odontology, “Iuliu Haţeganu” University of Medicine and Pharmacy of Cluj-Napoca, 400012 Cluj-Napoca, Romania; dan.pop@umfcluj.ro (D.P.); doina.rotaru@umfcluj.ro (D.-I.R.); 8 Department of Prosthetics and Dental Materials, “Iuliu Haţeganu” University of Medicine and Pharmacy of Cluj-Napoca, 400012 Cluj-Napoca, Romania; ioana.duncea@umfcluj.ro; 9 Department of Science and Materials Engineering, Technical University of Cluj-Napoca, 400114 Cluj-Napoca, Romania; mariana.pop@ipm.utcluj.ro; 10 Department of Road Vehicles and Transport, Technical University of Cluj-Napoca, 400114 Cluj-Napoca, Romania; aurel.chereches@auto.utcluj.ro; 11 Department of Motor Performance, Transylvania University of Brașov, 500036 Brașov, Romania; mindrescu.veronica@unitbv.ro

**Keywords:** dental implant, buckling bar, rigid medium, elastic medium, transfer-matrix, state vector, 00A69

## Abstract

Oral health is a very important issue today. This approach presents an original idea: to model the dental implant as a double-articulated buckling bar on an elastic environment. The mandibular bone is considered as the elastic environment. The buckling bar is analyzed using the Transfer-Matrix Method. The risk of buckling is higher for straight bars subjected to axial compression. Therefore, knowing the critical buckling force is very important, especially in the case of dental implants. This study, based on the Transfer-Matrix Method, was carried out in two steps. In the first step, a double-articulated buckling bar on a rigid environment is considered. The second step involves studying the same doubly articulated bar, but with the joint at the lower end resting on an elastic environment. The bone in which the implant is placed is considered as this elastic environment. The Transfer-Matrix Method is easy to implement and provides quick results for problems involving the shape optimization of structural components. This article presents a completely new idea and an original approach to buckling analysis, with applications to dental implants. This work will serve as a foundation for future research involving experimental investigations of dental implants.

## 1. Introduction

Implantology has recently taken a significant step forward, becoming one of the preferred solutions for both dentists and patients. Numerous studies have been developed regarding the methods and materials used in dental implants. This work presents an original approach to the study of a dental implant subjected to a vertical axial compression force. The analysis was performed using the Transfer-Matrix Method (TMM), taking advantage of the mathematical formalism offered by the Dirac and Heaviside functions and operators. The originality of this work lies in modeling the dental implant as a straight bar articulated at both ends.

The mathematical foundations of the TMM are presented in Ref. [1]. The TMM is based on matrix computation (operations with matrices and vectors), which is easy to program for iterative problems. Through this approach, we propose its application in the field of dental restorations using dental implants. The TMM is an elegant and relatively easy-to-apply numerical method for practical problems that require quick, immediate results. The TMM is a computational method that, based on a mathematical formalism introduced by Gery and Calgaro, as in Ref. [1], enables the determination of deformations and efforts in various structural elements as a function of their geometry, the material they are made of, and the loads to which they are subjected.

In our work, Section 2.2.1 presents the TMM applied to a bar subjected to buckling on a rigid foundation; the results are shown in Section 3.1 and validated against those obtained using the classical Strength of Materials approach from Refs. [2,3]. In Section 2.2.2, the TMM modeling of a bar subjected to buckling on an elastic foundation is presented—this model was used to simulate a dental implant, with the results shown in Section 3.2.

One of the key novelties of our study lies in the conceptual assimilation of the dental implant to a double-articulated bar subjected to buckling analysis. This mechanical analogy allows for a more precise understanding of the implant’s behavior under compressive forces. A second important contribution lies in the modeling of the mandibular bone as an elastic medium in which the dental implant is fixed. This approach reflects the biomechanical environment more realistically and enables a more accurate evaluation of the implant–bone interaction. Finally, a third original aspect of our research is the application of the TMM as the principal analytical tool for investigating both scenarios.

The idea of considering the mandibular or maxillary bone as an elastic environment for the dental implant is entirely new and original. Modeling the dental implant as a bar subjected to buckling on an elastic medium is also a novel approach. The TMM is easy to implement and provides fast results, especially for problems involving the shape optimization of structural components, such as dental implants.

Our research plan is more extensive and consists of several stages. The first stage is represented by this approach, which establishes the theoretical formulas for calculating straight bars on an elastic medium subjected to buckling. In the second stage, we will develop a calculation program based on the theoretical formulas established in this paper, which we have validated using classical Strength of Materials calculus from Refs. [2,3]. The program will allow input of the mechanical properties of the material, the geometric shape, and the loads to which the dental implant is subjected. In the third stage, we will validate the results obtained with our program by comparing them with results obtained through modeling the same real parts—in this case, actual dental implants conforming to ISO 14801, [4], using the Finite Element Method (FEM). The fourth stage will involve attaching to our calculus program an optimization module for the constructive shape of the real dental implant and cost optimization according to various criteria: the specific conditions identified by the doctor during the specialized consultation (including all risks and patient sensitivities); the doctor’s therapeutic recommendations; and the patient’s preferences (or restrictions) regarding material, cost, etc. The fifth stage will include experimental measurements on real dental implants, which will be compared with the results obtained by modeling with the two numerical methods, the TMM and the FEM.

Practical notions for dental preparation for fixed single-dental prostheses are given by the authors in Ref. [5]. Figures of graph partitioning by counting, sequence and layer matrices are presented in Ref. [6]. A systematic review of the effectiveness of cold atmospheric plasma (CAP) on bacterial reduction in dental implants is given by the authors in Ref. [7]. Ref. [8] gives a systematic review of and meta-analysis on the biological oriented preparation technique (BOPT) for tooth preparation. Research progress on the preparation process and material structure of 3D-printed dental implants, and their clinical applications, are given in Ref. [9]. In Ref. [10], the in situ growth of Mg-Fe layered double hydroxide (LDH) film on titanium dental implant substrates for pH regulation in oral environments is presented.

The materials used for dental implants play a crucial role in their performance. Among the many available materials, titanium and its alloys are the most important due to their durability and corrosion resistance. However, their most significant characteristic is biocompatibility, along with mechanical properties similar to bone tissue, such as high hardness and excellent corrosion resistance. Current knowledge and open questions about osseointegration of titanium, titanium alloy and zirconia dental implants are presented by the authors in Ref. [11]. A histomorphometry study in miniature pigs about the influence of surface characteristics on the bone integration of titanium implants is given in Ref. [12]. The authors of Ref. [13] present a recent development in beta titanium alloys for biomedical applications. The factors of influence and evaluation for the role of primary stability for successful osseointegration of dental implants can be seen in Ref. [14].

In Ref. [15] titanium-based biomaterials for preventing stress shielding between implant devices and bones are shown. The influence of implant taper on the primary and secondary stability of osseous-integrated titanium implants is presented in Ref. [16]. A literature review about the influence of thread geometry on implant osseointegration under immediate loading is presented in Ref. [17]. A review of the surface modifications of titanium alloys for biomedical applications can be seen in Ref. [18].

Relevant aspects in relation to the surface properties in titanium dental implants for cellular viability are given by the authors in Ref. [19]. The fabrication and properties of functionally graded dental implants can be found in Ref. [20]. A review about the revitalizing concepts with tapered implant technology in dentistry is presented by the authors in Ref. [21]. The authors of Ref. [22] give 5-year follow-up to soft-tissue augmentation around dental implants with a Connective Tissue Graft (CTG) and Xenogeneic Collagen Matrix (CMX).

The production and characterization of a bone, like porous Ti/Ti-hydroxyapatite functionally graded material, are discussed in Ref. [23]. Regarding dental implants, the modern materials and methods of their surface modification can be shown in Ref. [24].

Because the risk of buckling is higher in straight bars subjected to axial compression, it is essential to determine the critical buckling force. Theoretical and numerical studies of elastic buckling and load resistance of a shuttle-shaped double-restrained buckling-restrained brace is presented by the authors in Ref. [25]. The precise TMM for the elastic-buckling analysis of a compression bar can be shown in Ref. [26]. The buckling of piles in layered soils using the TMM is presented by the authors in Ref. [27]. A high order finite strip TMM for buckling analysis of single-branched cross-section thin-walled members can be seen in Ref. [28]. Biomechanical analysis of alveolar bone stress around implants with different thread designs and pitches in the mandibular molar area is given in Ref. [29].

The mechanical reliability and fatigue strength and a survival analysis of new polycrystalline translucent zirconia ceramics for monolithic restorations are given by the authors in Ref. [30]. Ref. [31] presents a study of bending beams on an elastic environment by the TMM. A biomechanical and histological analysis effect of implant thread geometry on secondary stability, bone density and bone-to-implant contact can be found in Ref. [32]. An in vitro study about the investigation of the influence of roughness and dental implant design on primary stability via an analysis of insertion torque and implant stability quotient is presented by the authors in Ref. [33].

Research on dental implants is extensive, as evidenced by the numerous publications in the field. Some of these, from both the technical and medical domains, are presented in the following.

The TMM is used for dental bridge calculus: the calculus of a monobloc dental bridge with two poles and two missing teeth as a beam using the TMM is presented in Ref. [34]. An approach using the TMM for mandible body bone calculus can be shown in Ref. [35], and calculus through the TMM of a beam with intermediate support with applications 34n dental restorations is given in Ref. [36]. An in vitro study about the fracture resistance of CAD/CAM provisional crowns with two different designs is presented in Ref. [37]. A structural stiffness matrix-based computational mechanics method of epithelial monolayers is given by the authors in Ref. [38].

The TMM is applied in many other engineering fields. The TMM for the calculus of a long cylindrical tube with industrial applications is shown in Refs. [39,40]. A three-periodic, chiral, tensegrity structure auxetic is presented by the authors in Ref. [41]. Multilevel structural defect-induced elastic wave tunability and localization of a tensegrity metamaterial can be shown in Ref. [42].

The TMM is applied in many engineering fields (as seen in [25,26,27,31,39,40,42,43]).

In Ref. [44], an insight into the dynamic mechanical property’s incorporation of disposed face mask to cement mortar material is presented. Research about giant elastic-wave asymmetry in a linear passive circulator can be found in Ref. [45]. Attenuation mechanisms of ultralow-frequency seismic metamaterials via complex band structure analysis can be shown in Ref. [46]. The friction-induced PTFE coating on dental restorative resin with ultralow friction and wear is presented in Ref. [47]. Research about the optimization of the multistage femtosecond laser drilling process using machine learning coupled with molecular dynamics is in Refs. [48,49] relates to the optimization of low-power femtosecond laser trepan drilling by machine learning and a high-throughput multi-objective genetic algorithm.

A robust predefined-time convergence zeroing neural network for dynamic matrix inversion is given in Refs. [50,51] and presents a fixed-time convergent and noise-tolerant zeroing neural network for online solution of time-varying matrix inversion. The Monte Carlo Method, used for the assessment of radiation attenuation properties in dental implants, is given in Ref. [52]. In Ref. [53] a retrospective study with a median follow-up of 17 months about the soft-tissue volume augmentation at dental implant placement using a collagen-based matrix characterized by oriented open pore structure is presented. A human-based study in providing a clinical analysis of the influence of surface roughness in the primary stability and osseointegration of dental implants can be found in Ref. [54]. Research about the case of Ti and ZrO_2_ for improving the clinical performance of dental implants through advanced surface treatments is in Ref. [55].

This work will serve as a foundation for future research on dental implants. The TMM is easy to implement and suitable for theoretical simulations aimed at optimizing implant shapes. In the future, these theoretical results can be validated using the FEM and through experimental tests, both in the laboratory and in situ.

## 2. Materials and Methods

### 2.1. Materials

As is well known, a dental implant consists of a dental abutment and a crown that replaces the missing tooth. The abutment has its lower end embedded in the bone, while the upper end serves as the base for the dental crown, which is secured by a screw. This screw, placed into the bone, replaces the dental root and acts as a connector between the bone and the dental crown. Several types of materials can be used for a dental abutment, with titanium and zirconium being the most commonly used ones. Titanium is particularly important because of its durability and resistance to corrosion. Its most significant characteristic is biocompatibility, which makes it especially suitable for areas subject to high masticatory forces, such as the back of the mouth.

Zirconium is highly valued from an esthetic point of view. It integrates well with gingival tissues and is particularly used in areas where esthetics are important, such as the front part of the mouth. Gold and its alloys are less preferred today due to their high cost and inappropriate color, even though they are biocompatible and easy to process. Poly-ether-ether-ketone (PEEK) is biocompatible and has good elasticity. It is an advanced plastic material used in special situations or for temporary dental work.

To achieve an efficient and versatile solution, a hybrid version can be used. There is also the possibility of creating a hybrid implant, combining the advantageous characteristics of two or more materials to obtain the optimal solution, both from an esthetic and functional perspective. In this paper, a straight implant is studied, modeled as a double-articulated bar subjected to buckling by an axial compression force.

### 2.2. Methods

#### 2.2.1. The Transfer-Matrix Method for a Doubly Articulated Straight Bar on a Rigid Environment

The basis of the TMM is presented in Ref. [1]. To study a doubly articulated bar subject to buckling on an elastic environment, the bar will first be studied in a rigid environment.

##### The Differential Equation of the Deformed Average Fiber for a Doubly Articulated Straight Bar Subjected to Buckling in a Rigid Environment

**Figure 1:**
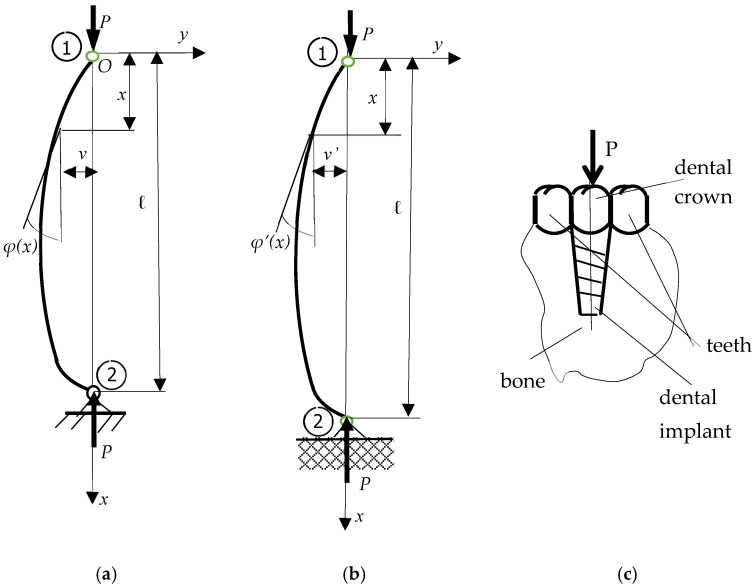
A doubly articulated buckling straight bar and a model of dental implant: (**a**) A doubly articulated buckling straight bar on a rigid environment; (**b**) a double-articulated buckling bar on an elastic environment; (**c**) a dental implant modeled as a double-articulated buckling bar on an elastic environment.

Several working hypotheses are used to deduce the differential equation of the deformed average fiber for a doubly articulated straight bar subjected to buckling, as shown in Figure 1a. The working hypotheses are:

- The bar is lengthened by *l*;
- The bar has a constant moment of inertia along its entire length;
- A force P acts along the *x*-axis of the bar at its extremities, which tend to compress the bar, as shown in Figure 1a;
- At a section *x* the deflection is denoted by *v*.

- At a section *x*, the rotation is denoted by *φ*;
- The curvature of the deformed elastic line is relatively small.

The differential equation of the deformed average fiber, according to Ref. [2], is given by (1):

(1) $$EI\frac{d^{2}v}{dx^{2}}=-M\left(x\right),$$

*M*(*x*) is the total bending moment at the current section *x*, given by the Expression (2):

(2) $$M\left(x\right)=Pv,$$

Using Expression (2), the differential Equation (1) becomes (3):

(3) $$EI\frac{d^{2}v}{dx^{2}}=-Pv,$$

By differentiating (3) twice, it is successively obtained (4):

(4) $$EI\frac{d^{3}v}{dx^{3}}=-P\frac{dv}{dx},$$

and (5):

(5) $$EI\frac{d^{4}v}{dx^{4}}=-P\frac{d^{2}v}{dx^{2}},$$

or, (6):

(6) $$EI\frac{d^{4}v}{dx^{4}}+P\frac{d^{2}v}{dx^{2}}=0,$$

(6) is a homogeneous linear differential equation with constant coefficients, expressed as (7):

(7) $$\beta^{2}=\frac{P}{EI},$$

where *E* is Young’s modulus, and *I* is the moment of inertia of the bar’s cross-section.

Using (7), the differential Equation (6) becomes (8):

(8) $$\frac{d^{4}v}{dx^{4}}+\beta^{2}\frac{d^{2}v}{dx^{2}}=0,$$

with the characteristic Equation (9):

(9) $$r^{4}+\beta^{2}r^{2}=0,$$

The general solution of the characteristic equation (9) is of the form given in (10):

(10) $$v\left(x\right)=B_{1}\;cos\;\beta x+B_{2}\sin\beta x+B_{3}x+B_{4},$$

For *x* = 0, (Figure 1a) the result was as follows (11):

(11) $$v_{0}=B_{1}+B_{4},$$

The relation (10) is differentiated once w.r.t. *x* and the Expression (12) is obtained:

(12) $$\varphi \left(x\right)\;=\;\frac{dv}{dx}={-B}_{1}\;\beta \sin\beta x+B_{2}\;\beta \cos\beta x+B_{3},$$

For *x* = 0, the result was as follows (13):

(13) $$\varphi_{0}=B_{2\;}\beta +B_{3},$$

The relation (12) is differentiated once w.r.t. *x* and the Expression (14) is obtained:

(14) $$\frac{M\left(x\right)}{EI}\;=\;\frac{\;d^{2}v}{{dx}^{2}}={-B}_{1\;}\beta^{2}\cos\beta x{-B}_{2}\;\beta^{2}\sin\beta x,$$

For *x* = 0, the result was as follows (15):

(15) $$M_{0}=-B_{1}\;EI\;\beta^{2},$$

According to relation (1), Expression (14) can be written as (16):

(16) $$\frac{\;d^{2}v}{{dx}^{2}}=-\frac{M\left(x\right)}{EI}=B_{1\;}\beta^{2}\cos\beta x{+B}_{2}\;\beta^{2}\sin\beta x,$$

According to the differential relationships between internal forces after Ref. [2]:

(17) $$\frac{dM}{dx}=T,$$

So, by differentiating relation (16) once, we obtain (18):

(18) $$\frac{\;d^{3}v}{{dx}^{3}}=\frac{1}{EI}\frac{dM\left(x\right)}{dx}=\;\frac{T\left(x\right)}{EI}{=B}_{1\;}\beta^{3}\sin\beta x-B_{2}{\;\beta }^{3}\cos\beta x,$$

For *x* = 0, the result was as follows (19):

(19) $$T_{0}=-B_{2}\;EI\;\beta^{3},$$

The index 0 refers to the origin section for *x* = 0, which is at the upper end of the bar. This means that the deformations *v*_0_ and *φ*_0_, as well as the efforts *M*_0_ and *T*_0_, are defined. in section 0.

The four integration constants *B_i_*, where *i* = 1, 4, are calculated, and the Expressions (20)–(23) are obtained:

(20) $$B_{1}=-\frac{M_{0}}{EI{\;\beta }^{2}},$$

(21) $$B_{2}=-\frac{T_{0}}{EI{\;\beta }^{3}},$$

(22) $$B_{3}=\beta_{0}+\frac{T_{0}}{EI{\;\beta }^{2}},$$

(23) $$B_{4}=v_{0}+\frac{M_{0}}{EI{\;\beta }^{2}},$$

With the four integration constants B_i_, where i = 1, 4:

- Expression (10) becomes (24):

(24) $$v\left(x\right)=\left(-\frac{M_{0}}{EI\beta^{2}}\right)cos\;\beta x+\left(-\frac{T_{0}}{\mathrm{EI}\beta^{3}}\right)\sin\beta x+\left(\varphi_{0}+\frac{T_{0}}{EI\beta^{2}}\right)x+\left(v_{0}+\frac{M_{0}}{EI\beta^{2}}\right),$$

or, (25):

(25) $$v\left(x\right)=v_{0}+{x\varphi }_{0}+\frac{1-\cos\beta x}{EI\beta^{2}}M_{0}+\frac{\beta x-sin\beta x}{EI\beta^{3}}T_{0},$$

- Expression (11) becomes (26):

(26) $$\varphi \left(x\right)=\left(\frac{M_{0}}{EI\beta^{2}}\right)\beta \sin\beta x+\left(-\frac{T_{0}}{EI\beta^{3}}\right)\beta \cos\beta x+\left(\varphi_{0}-\frac{T_{0}}{EI\varphi^{2}}\right),$$

or, (27):

(27) $$\;\;\varphi \left(x\right)=\varphi_{0}-\frac{sin\;\varphi x}{EI\varphi }M_{0}-\frac{cos\;\varphi x+1}{EI\beta^{2}}T_{0},$$

- Expression (13) becomes (28):

(28) $$M\left(x\right)=-\left(-\frac{M_{0}}{EI\beta^{2}}\right)\beta^{2}\cos\beta x-\left(-\frac{T_{0}}{EI\beta^{3}}\right)\beta^{2}\sin\beta x$$

or, (29):

(29) $$M\left(x\right)=\frac{cos\;\beta x}{EI}M_{0}+\frac{sin\;\beta x}{EI\beta }T_{0},$$

- Expression (15) becomes (30):

(30) $$T\left(x\right)=-\left(-\frac{M_{0}}{EI\beta^{2}}\right)\beta^{3}\sin\beta x+\left(-\frac{T_{0}}{EI\beta^{3}}\right)\beta^{3}\cos\beta x,$$

or, (31):

(31) $$T\left(x\right)=+\frac{\beta \;sin\;\beta x}{EI}M_{0}-\frac{\cos\beta x}{EI}T_{0}.$$

##### The Transfer Matrix for a Doubly Articulated Buckling Bar (Figure 1a)

The state vectors and the transfer-matrix of a straight bar, loaded with a concentrated force at both ends, as shown in Figure 1a, can be defined.

The deformation *y* can be written in section *x*.

- The state vector for section *x* of the bar

It can be defined as the state vector at section *x* of a bar, a column vector with four elements, as shown in (32):

(32) $$\left\{V\left(x\right)\right\}={\left\{V\right\}}_{x}=\;{\left\{v\left(x\right),\;\varphi \left(x\right),\;M\left(x\right),\;T\left(x\right)\right\}}^{-1},$$

when:

- {*V*(*x*)} = {*V*}*_x_* is the state vector corresponding to section *x*;
- *v*(*x*) is the displacement corresponding to section *x*;
- *φ*(*x*) is the rotation corresponding to section *x*;
- *M*(*x*) is the bending moment corresponding to section *x*;
- *T*(*x*) is the shear force corresponding to section *x*.

For the first section, the left section (the origin, for *x* = 0), the state vector is given by (33):

(33) $$\;\left\{V\left(0\right)\right\}={\left\{V\right\}}_{0}=\;{\left\{v\left(0\right),\;\varphi \left(0\right),\;M\left(0\right),\;T\left(0\right)\right\}}^{-1}={\left\{v_{0},\;\varphi_{0},\;M_{0},T_{0}\right\}}^{-1},$$

At the last section, on the right end, for *x = l*, the state vector can be written as (34)

(34) $$\left\{V\left(l\right)\right\}={\left\{V\right\}}_{l}={\left\{v\left(l\right),\;\varphi \left(l\right),\;M\left(l\right),\;T\left(l\right)\right\}}^{-1}={\left\{v_{l},\;\varphi_{l},\;M_{l},T_{l}\right\}}^{-1},$$

- The Transfer-Matrix relation for a straight bar

The connection between the origin section (*x* = 0) and a certain section *x* can be made through the matrix relation (35):

(35) $${\left\{V\right\}}_{x}=\;{\left[T\right]}_{x}{\cdot \left\{V\right\}}_{0}\;{+\left[T_{e}\right]}_{x}{\cdot \left\{V_{e}\right\}}_{x},$$

with notation:

- {*V*}*_x_* is the state vector corresponding to section *x*;
- [*T*]*_x_* is the transfer matrix between the origin and section *x*;
- {*V*}_0_ is the state vector corresponding to the origin section;
- [T_e_]_x_ · {V_e_}_x_ is a term that corresponds to the external loads acting on section *x*.

For section *x*, Expressions (25), (27), (29), and (31) can be written as (36):

(36) $$\left\{\begin{array}{c} v\left(x\right)=v_{0}+{x\varphi }_{0}+\frac{1-\cos\beta x}{EI\beta^{2}}M_{0}+\frac{\beta x-sin\beta x}{EI\beta^{3}}T_{0}\;\;\;\;\\ \varphi \left(x\right)=\varphi_{0}-\frac{sin\;\beta x}{EI\beta }M_{0}-\frac{cos\;\beta x+1}{EI\beta^{2}}T_{0\;\;}\\ M\left(x\right)=\frac{cos\;\beta x}{EI}M_{0}+\frac{sin\;\beta x}{EI\beta }T_{0}\\ \;T\left(x\right)=+\frac{\beta \;sin\;\beta x}{EI}M_{0}-\frac{\cos\beta x}{EI}T_{0} \end{array}\right.,$$

or, in the form of a matrix relation as given in (37):

(37) $${\left\{V\right\}}_{x}=\left\{\begin{array}{c} v\left(x\right)\\ \varphi \left(x\right)\\ M\left(x\right)\\ T\left(x\right) \end{array}\right\}=\;\left[\begin{array}{cccc} 1 & x & \frac{1-\cos\beta x}{EI\beta^{2}} & \frac{\beta x-sin\beta x}{EI\alpha^{3}}\\ 0 & 1 & -\frac{sin\;\beta x}{EI\beta } & -\frac{cos\;\beta x+1}{EI\alpha^{2}}\\ 0 & 0 & \frac{cos\;\beta x}{EI} & +\frac{sin\;\beta x}{EI\beta }\\ 0 & 0 & +\frac{\beta \;sin\;\beta x}{EI} & -\frac{\cos\beta x}{EI} \end{array}\right]\left\{\begin{array}{c} v_{0}\\ \varphi_{0}\\ M_{0}\\ T_{0} \end{array}\right\},$$

For the last section, where *x* = *l*, the matrix relation (37) can be written as (38):

(38) $$\left\{\begin{array}{c} v\left(l\right)\\ \varphi \left(l\right)\\ M\left(l\right)\\ T\left(l\right) \end{array}\right\}=\;\left[\begin{array}{cccc} 1 & l & \frac{1-\cos\beta l}{EI\beta^{2}} & \frac{\beta l-sin\beta l}{EI\beta^{3}}\\ 0 & 1 & -\frac{sin\;\beta l}{EI\beta } & -\frac{cos\;\beta l+1}{EI\beta^{2}}\\ 0 & 0 & \frac{cos\;\beta l}{EI} & +\frac{sin\;\beta l}{EI\beta }\\ 0 & 0 & +\frac{\beta \;sin\;\beta l}{EI} & -\frac{\cos\beta l}{EI} \end{array}\right]\left\{\begin{array}{c} v_{0}\\ \varphi_{0}\\ M_{0}\\ T_{0} \end{array}\right\},$$

Now, it is possible to apply the boundary conditions at both ends of the doubly articulated straight bar, as shown in (39):

(39) $$\;\;\;\;\;\left\{\begin{array}{c} v_{0}=0\\ M_{0}=0\\ v\left(l\right)=0\\ M\left(l\right)=0 \end{array}\right.,$$

#### 2.2.2. The Transfer-Matrix Method for a Straight Doubly Articulated Buckling Bar on an Elastic Environment

The aim is to derive the Transfer Matrix for a bar placed on a perfectly uniform and homogeneous elastic medium, given the function representing the elasticity coefficient of the medium, *k*(*x*), (Figure 1b).

All terms marked with an apostrophe (‘) refer to the bar on the elastic medium.

##### The Differential Equation for the Deformed Average Fiber of a Doubly Articulated Straight Bar Subjected to Buckling on an Elastic Medium

- Average deformed fiber of a bar resting on an elastic medium

The dental implant is modeled as an articulated bar on an elastic medium at the bottom (with the bone considered as the elastic medium) and articulated at the top. The goal is to derive the Transfer Matrix for a bar on an elastic medium. The elastic medium can be uniform and homogeneous. It is necessary to know the function for the elasticity coefficient of the elastic medium, *k*(*x*).

A working hypothesis was considered, stating that the deflections at both ends of the bar are zero.

Following the analogy with relation (6) for a bar on a rigid environment, the corresponding differential equation for a bar on an elastic medium can be expressed as (40):

(40) $$EI\frac{d^{4}v\prime }{dx^{4}}+P\frac{d^{2}v\prime }{dx^{2}}+kv^{\prime }=0,$$

(40) is a homogeneous linear differential equation of order 4. It is denoted as (41):

(41) $$\;2c=\frac{P}{EI\;\;},$$

and as (42):

(42) $$\gamma =\frac{k}{EI},$$

For the differential equation of the deformed average fiber (40), the corresponding characteristic equation is (43):

(43) $$r^{4}+2cr^{2}+\gamma =0,$$

with the determinant defined by (44):

(44) $$\Delta =c^{2}-\gamma ,$$

There is no solution for (45):

(45) $$\;c^{2}<\gamma ,$$

If (46):

(46) $$c^{2}>\gamma ,$$

according to Ref. [1], relations (47) can be written as:

(47) $$\left\{\begin{array}{c} \beta^{2}=c+\sqrt{\Delta }\\ \delta^{2}=c-\sqrt{\Delta } \end{array}\right.,$$

Thus, the general solution of the differential equation is given by (48):

(48) $$v\prime \left(x\right)=B_{1}^{\prime }\;cos\;\beta x+B_{2}^{\prime }\sin\beta x+B_{3}^{\prime }\;cos\;\delta x+B_{4}^{\prime }\;sin\;\delta x,$$

Expression (48) is differentiated once w.r.t. *x*, and Expression (49) is obtained:

(49) $$\varphi^{\prime }\left(x\right)=\;\frac{dv\prime }{dx}={-B}_{1}^{\prime }\;\beta \sin\beta x+B_{2}^{\prime }\;\beta \cos\beta x-B_{3}^{\prime }\;\delta \;sin\;\delta x+B_{4\;}^{\prime }\delta \;cos\;x,$$

Expression (49) is differentiated once w.r.t. *x*, and Expression (50) is obtained for the moment:

(50) $$\frac{M^{\prime }\left(x\right)}{EI}=\;\;\frac{d^{2}v\prime }{dx^{2}}=-B_{1}^{\prime }{\;\beta }^{2}\cos\beta x-B_{2}^{\prime }\;\beta^{2}\sin\beta x-B_{3}^{\prime }\;\delta^{2}\;cos\;\delta x-B_{4\;}^{\prime }\delta^{2}\;sin\;\delta x,$$

Expression (50) is differentiated once w.r.t. *x*, and Expression (51) is obtained for the cutting force:

(51) $$\frac{T^{\prime }\left(x\right)}{EI}=\frac{1}{EI}\frac{{dM}^{\prime }\left(x\right)}{dx}=\frac{d^{3}v^{\prime }}{dx^{3}}={+B}_{1\;}^{\prime }\beta^{3}\sin\beta x-B_{2}^{\prime }{\;\beta }^{3}\cos\beta x+B_{3}^{\prime }\;\delta^{3}\;sin\;\delta x-B_{4}^{\prime }\;\delta^{3}\;cos\;\delta x,$$

For *x* = 0, i.e., at the origin, Expressions (48), (49), (50), and (51) reduce to (52), (53), (54), and (55), respectively:

(52) $$v_{0}^{\prime }=B_{1}^{\prime }+B_{3}^{\prime },$$

(53) $$\varphi_{0}^{\prime }=B_{2}^{\prime }\;\beta +B_{4}^{\prime }\;\delta ,$$

(54) $$M_{0}^{\prime }=-B_{1}^{\prime }\;EI\beta^{2}-B_{3}^{\prime }\;EI\delta^{2},$$

(55) $$T_{0}^{\prime }=-B_{2}^{\prime }\;EI\beta^{3}-B_{4}^{\prime }\;EI\delta^{3},$$

The four integration constants $B_{1}^{\prime }$, *i* = 1, 4, are calculated, and Expressions (56)–(59) are obtained:

(56) $$B_{1}^{\prime }=-\frac{1}{2\sqrt{\Delta }}\left(\delta^{2}v_{0}^{\prime }+\frac{M_{0}^{\prime }}{EI}\right),$$

(57) $$B_{2}^{\prime }=-\frac{1}{2\beta \sqrt{\Delta }}\left(\delta^{2}\varphi_{0}^{\prime }+\frac{T_{0}^{\prime }}{EI}\right),$$

(58) $$B_{3}^{\prime }=\frac{1}{2\sqrt{\Delta }}\left(\beta^{2}v_{0}^{\prime }+\frac{M_{0}^{\prime }}{EI}\right),$$

(59) $$B_{4}^{\prime }=\frac{1}{2\delta \sqrt{\Delta }}\left(\beta^{2}\varphi_{0}^{\prime }+\frac{T_{0}^{\prime }}{EI}\right),$$

With the four integration constants $B_{1}^{\prime }$, where i = 1, 4, we obtain:

- Expression (48) becomes (60):

(60) $$v^{\prime }(x)=\left[-\frac{1}{2\sqrt{\Delta }}\left(\delta^{2}v_{0}^{\prime }+\frac{M_{0}^{\prime }}{EI}\right)\right]\;cos\;\beta x+\left[-\frac{1}{2\beta \sqrt{\Delta }}\left(\delta^{2}\varphi_{0}^{\prime }+\frac{T_{0}^{\prime }}{EI}\right)\right]\sin\beta x+,\;+\left[\frac{1}{2\sqrt{\Delta }}\left(\beta^{2}v_{0}^{\prime }+\frac{M_{0}^{\prime }}{EI}\right)\right]\cos\delta x+\left[\frac{1}{2\delta \sqrt{\Delta }}\left(\beta^{2}\varphi_{0}^{\prime }+\frac{T_{0}^{\prime }}{EI}\right)\right]\;sin\;\delta x$$

or, (61):

(61) $$v^{\prime }\left(x\right)=\frac{\beta^{2}cos\;\delta x-\delta^{2}cos\;\beta x}{2\sqrt{\Delta }}v_{0}^{\prime }-\frac{\delta^{3}\sin\beta x-\beta^{3}\sin\delta x}{2\beta \delta \sqrt{\Delta }}-\frac{cos\;\beta x-cos\;\delta x}{2EI\sqrt{\Delta }}M_{0}^{\prime }-\frac{\delta \sin\beta x-\beta \sin\delta x}{2\beta \delta EI\sqrt{\Delta }}T_{0}^{\prime },$$

- Expression (49) becomes (62):

(62) $$\varphi^{\prime }\left(x\right)=+\frac{1}{2\sqrt{\Delta }}\left(\delta^{2}v_{0}^{\prime }+\frac{M_{0}^{\prime }}{EI}\right)\;\beta \sin\beta x-\frac{1}{2\beta \sqrt{\Delta }}\left(\delta^{2}\varphi_{0}^{\prime }+\frac{T_{0}^{\prime }}{EI}\right)\beta \cos\beta x-\frac{1}{2\sqrt{\Delta }}\left(\beta^{2}v_{0}^{\prime }+\frac{M_{0}^{\prime }}{EI}\right)\delta sin\delta x+\frac{1}{2\delta \sqrt{\Delta }}\left(\beta^{2}\varphi_{0}^{\prime }+\frac{T_{0}^{\prime }}{EI}\right)\delta cos\delta x,$$

or, (63):

(63) $$\varphi^{\prime }\left(x\right)=\frac{\beta \delta \left(\delta \;sin\;\beta x-\beta \;sin\;\delta x\right)}{2\sqrt{\Delta }}v_{0}^{\prime }-\frac{\delta^{2}\cos\beta x-\beta^{2}\cos\delta x}{2\sqrt{\Delta }}\varphi_{0}^{\prime }+\frac{\beta \;sin\;\beta x-\delta sin\;\delta x}{2EI\sqrt{\Delta }}M_{0}^{\prime }-\frac{cos\;\beta x-cos\;\delta /x}{2EI\sqrt{\Delta }}T_{0}^{\prime },$$

- Expression (50) becomes (64):

(64) $$M^{\prime }\left(x\right)=\frac{EI}{2\sqrt{\Delta }}\left(\delta^{2}v_{0}^{\prime }+\frac{M_{0}^{\prime }}{EI}\right){\;\beta }^{2}\cos\beta x+\frac{EI}{2\beta \sqrt{\Delta }}\left(\delta^{2}\varphi_{0}^{\prime }+\frac{T_{0}^{\prime }}{EI}\right)\;\beta^{2}\sin\beta x-\frac{EI}{2\sqrt{\Delta }}\left(\beta^{2}v_{0}^{\prime }+\frac{M_{0}^{\prime }}{EI}\right)\;\delta^{2}\cos\delta x\;-\frac{EI}{2\delta \sqrt{\Delta }}\left(\beta^{2}\varphi_{0}^{\prime }+\frac{T_{0}^{\prime }}{EI}\right)\delta^{2}\;sin\;\delta x$$

or, (65):

(65) $$M^{\prime }\left(x\right)=+\frac{\beta^{2}\delta^{2}EI\left(\cos\beta x-cos\;\delta x\right)}{2\sqrt{\Delta }}v_{0}^{\prime }+\frac{\beta \delta EI\left(\delta \sin\beta x-\mathrm{\beta sin}\delta x\right)}{2\sqrt{\Delta }}\varphi_{0}^{\prime }+\frac{\beta^{2}cos\;\beta x-\delta^{2}\;cos\;\delta x}{2\sqrt{\Delta }}M_{0}^{\prime }+\frac{\beta \sin\beta x\;-\delta \sin\delta x}{2\beta \delta \sqrt{\Delta }}T_{0}^{\prime },$$

- Expression (52) becomes (66):

(66) $$\;T^{\prime }\left(x\right)=EI[-\frac{1}{2\sqrt{\Delta }}\left(\delta^{2}v_{0}^{\prime }+\frac{M_{0}^{\prime }}{EI}\right)\beta^{3}\sin\beta x+\frac{1}{2\beta \sqrt{\Delta }}\left(\delta^{2}\varphi_{0}^{\prime }+\frac{T_{0}^{\prime }}{EI}\right){\;\beta }^{3}\cos\beta x+\frac{1}{2\sqrt{\Delta }}\left(\beta^{2}v_{0}^{\prime }+\frac{M_{0}^{\prime }}{EI}\right)\;\delta^{3}\;sin\;\delta x-\frac{1}{2\delta \sqrt{\Delta }}\left(\beta^{2}\varphi_{0}^{\prime }+\frac{T_{0}^{\prime }}{EI}\right)\;\delta^{3}\;cos\;\delta x],$$

or, (67):

(67) $$T^{\prime }\left(x\right)=-\frac{\beta^{2}\delta^{2}EI\left(\delta \;sin\;\delta x-\beta \;sin\;\beta x\right)}{2\sqrt{\Delta }}v_{0}^{\prime }+\frac{\beta^{2}\delta^{2}EI\left(\cos\beta x-cos\;\delta x\right)}{2\sqrt{\Delta }}\varphi_{0}^{\prime }+\frac{\delta^{3}\;sin\;\delta x-\beta^{3}\;sin\;\beta x}{2EI\sqrt{\Delta }}M_{0}^{\prime }+\frac{\beta^{2}\;cos\;\beta x-\delta^{2}\;cos\;\delta x}{2EI\sqrt{\Delta }}T_{0}^{\prime },$$

#### 2.2.3. Calculation of a Dental Implant Modeled as a Double-Articulated Buckling Bar Under Axial Compression, on an Elastic Environment, Using the TMM, (Figure 1c)

The dental implant is modeled as a double-articulated buckling bar subjected to axial compression, placed on an elastic medium at the bottom.

The state vectors and the transfer matrix of a straight bar loaded with a concentrated force at its ends can be defined, as shown in Figure 1c. In Figure 1c a dental implant modeled as a double-articulated buckling bar is presented.

In section *x*, the deformation *v* can be written.

- The state vector for a section

The state vector at section x of the bar can be defined as a column vector with four elements, as shown in (68):

(68) $${\left\{V\prime \right\}}_{x}=\;{\left\{v\prime \left(x\right),\;\varphi \prime \left(x\right),\;M\prime \left(x\right),\;T\prime \left(x\right)\right\}}^{-1},$$

when:

- {V′(x)} = {V′}_x_ is the state vector corresponding to section x;
- v′(x) is the deflection at section x;
- φ′(x) is the rotation at section x;
- M′(x) is the bending moment at section x;
- T′(x) is the shear force at section x.

For the first section, at the origin (x = 0), the state vector is given by (69):

(69) $$\left\{V\prime \left(0\right)\right\}={\left\{V\prime \right\}}_{0}=\;{\left\{v\prime \left(0\right),\varphi \;\prime \left(0\right),\;M\prime \left(0\right),\;T\prime \left(0\right)\right\}}^{-1}={\left\{v_{0}^{\prime },\;\varphi_{0}^{\prime },\;M_{0}^{\prime },T_{0}^{\prime }\right\}}^{-1},$$

At the last section, for x = l, the state vector can be written as (70):

(70) $$\left\{V\prime \left(l\right)\right\}={\left\{V\prime \right\}}_{l}=\;{\left\{v^{\prime }(l),\;\varphi^{\prime }(l),\;M^{\prime }(l),T\prime \left(l\right)\right\}}^{-1}={\left\{v_{l}^{\prime },\;\varphi_{l}^{\prime },\;M_{l}^{\prime },\;T_{l}^{\prime }\right\}}^{-1},$$

- The Transfer-Matrix relation for a double-articulated buckling bar on an elastic environment

The connection between the first section at *x* = 0 and a certain section *x* can be made through the matrix relation (71):

(71) $${\left\{V\prime \right\}}_{x}=\;{\left[T\prime \right]}_{x}{\cdot \left\{V\prime \right\}}_{0}\;{+\left[T_{e}^{\prime }\right]}_{x}{\cdot \left\{V_{e}^{\prime }\right\}}_{x},$$

with the following notations:

- {V′}_x_ is the state vector corresponding to section x;
- [T′]_x_ is the Transfer Matrix between the origin and section x;
- {V′}_0_ is the state vector corresponding to the origin (section 0);
- [T_e_′]_x_ · {V_e_′}_x_ is a term that corresponds to the external loads acting on section x.

For section *x*, Expressions (61), (63), (65) and (67) can be written as (72):

(72) $$\left\{\begin{array}{c} v^{\prime }\left(x\right)=\frac{\beta^{2}cos\;\delta x-\delta^{2}cos\;\beta x}{2\sqrt{\Delta }}v_{0}^{\prime }-\frac{\delta^{3}\sin\beta x-\beta^{3}\sin\delta x}{2\beta \delta \sqrt{\Delta }}\varphi_{0}^{\prime }-\frac{cos\;\beta x-cos\;\delta x}{2EI\sqrt{\Delta }}M_{0}^{\prime }-\frac{\mathrm{\delta sin}\beta x-\beta \sin\delta x}{2\beta \delta EI\sqrt{\Delta }}T_{0}^{\prime }\;\\ \;\;\varphi^{\prime }\left(x\right)=\frac{\beta \delta \left(\delta \;sin\;\beta x-\beta \;sin\;\delta x\right)}{2\sqrt{\Delta }}v_{0}^{\prime }-\frac{\delta^{2}\cos\beta x-\beta^{2}\cos\delta x}{2\sqrt{\Delta }}\varphi_{0}^{\prime }+\frac{\beta \;sin\;\beta x-\delta \;sin\;\delta x}{2EI\sqrt{\Delta }}M_{0}^{\prime }-\frac{cos\;\beta x-cos\;\delta x}{2EI\sqrt{\Delta }}T_{0}^{\prime }\\ M^{\prime }\left(x\right)=+\frac{\beta^{2}\delta^{2}EI\left(\cos\beta x-cos\;\delta x\right)}{2\sqrt{\Delta }}v_{0}^{\prime }+\frac{\beta \delta EI\left(\delta \sin\beta x-\beta \sin\delta x\right)}{2\sqrt{\Delta }}\varphi_{0}^{\prime }+\frac{\beta^{2}cos\;\beta x-\delta^{2}\;cos\;\delta x}{2\sqrt{\Delta }}M_{0}^{\prime }+\frac{\beta \sin\beta x\;-\delta \sin\delta x}{2\beta \delta \sqrt{\Delta }}T_{0}^{\prime }\\ \;T^{\prime }\left(x\right)=-\frac{\beta^{2}\delta^{2}EI\left(\delta \;sin\;\delta x-\beta \;sin\;\beta x\right)}{2\sqrt{\Delta }}v_{0}^{\prime }+\frac{\beta^{2}\delta^{2}EI\left(\cos\beta x-cos\;\delta x\right)}{2\sqrt{\Delta }}\varphi_{0}^{\prime }+\frac{\delta^{3}\;sin\;\delta x-\beta^{3}\;sin\;\beta x}{2EI\sqrt{\Delta }}M_{0}^{\prime }+\frac{\beta^{2}\;cos\;\beta x-\delta^{2}\;cos\;\delta x}{2EI\sqrt{\Delta }}T_{0}^{\prime } \end{array}\right.,$$

or, in the form of a matrix relation, as shown in (73):

(73) $$\;\;\;\left\{\begin{array}{c} v\prime \left(x\right)\\ \varphi \prime \left(x\right)\\ M\prime \left(x\right)\\ T\prime \left(x\right) \end{array}\right\}\left[\begin{array}{cccc} \frac{\beta^{2}cos\;\delta x-\delta^{2}cos\;\beta x}{2\sqrt{\Delta }} & -\frac{\delta^{3}\sin\beta x-\beta^{3}\sin\delta x}{2\beta \delta \sqrt{\Delta }} & -\frac{cos\;\beta x-cos\;\delta x}{2EI\sqrt{\Delta }} & -\frac{\mathrm{\delta sin}\beta x-\beta \sin\delta x}{2\beta \delta EI\sqrt{\Delta }}\\ \frac{\beta \delta \left(\delta \;sin\;\beta x+\beta \;sin\;\delta x\right)}{2\sqrt{\Delta }} & \frac{{\beta^{2}\cos\delta x-\delta }^{2}\cos\beta x}{2\sqrt{\Delta }} & +\frac{\beta sin\;\beta x-\delta \;sin\;\delta x}{2EI\sqrt{\Delta }} & -\frac{cos\;\beta x-cos\;\delta x}{2EI\sqrt{\Delta }}\\ +\frac{\beta^{2}\delta^{2}EI\left(\cos\beta x-cos\;\delta x\right)}{2\sqrt{\Delta }} & +\frac{\beta \delta EI\left(\delta \sin\beta x-\beta \sin\delta x\right)}{2\sqrt{\Delta }} & \frac{\beta^{2}cos\beta x-\delta^{2}\;cos\;\delta x}{2\sqrt{\Delta }} & -\frac{\delta \sin\delta x-\beta \sin\beta x\;}{2\sqrt{\Delta }}\\ \frac{\beta^{2}\delta^{2}EI\left(\beta \;sin\;\beta x-\delta \;sin\;\delta x\right)}{2\sqrt{\Delta }} & +\frac{\beta^{2}\delta^{2}EI\left(\cos\beta x-cos\;\delta x\right)}{2\sqrt{\Delta }} & -\frac{{\beta^{3}\;sin\;\beta x-\delta }^{3}\;sin\;\delta x}{2\sqrt{\Delta }} & \frac{\beta^{2}\;cos\;\beta x-\delta^{2}\;cos\;\delta x}{2\sqrt{\Delta }} \end{array}\right]\left\{\begin{array}{c} v_{0}^{\prime }\\ \varphi_{0}^{\prime }\\ M_{0}^{\prime }\\ T_{0}^{\prime } \end{array}\right\},$$

For the last section, at *x* = *l*, the matrix relation (73) can be written as (74):

(74) $$\left\{\begin{array}{c} v\prime \left(l\right)\\ \varphi \prime \left(l\right)\\ M\prime \left(l\right)\\ T\prime \left(l\right) \end{array}\right\}\left[\begin{array}{cccc} \frac{\beta^{2}cos\;\delta l-\delta^{2}cos\;\beta l}{2\sqrt{\Delta }} & -\frac{\delta^{3}\sin\beta l-\beta^{3}\sin\delta l}{2\beta \delta \sqrt{\Delta }} & -\frac{cos\;\beta l-cos\;\delta l}{2EI\sqrt{\Delta }} & -\frac{\mathrm{\delta sin}\beta l-\beta \sin\delta l}{2\beta \delta EI\sqrt{\Delta }}\\ \frac{\beta \delta \left(\delta \;sin\;\beta l+\beta \;sin\;\delta l\right)}{2\sqrt{\Delta }} & \frac{{\beta^{2}\cos\delta l-\delta }^{2}\cos\beta l}{2\sqrt{\Delta }} & +\frac{\beta sin\;\beta l-\delta \;sin\;\delta l}{2EI\sqrt{\Delta }} & -\frac{cos\;\beta l-cos\;\delta l}{2EI\sqrt{\Delta }}\\ +\frac{\beta^{2}\delta^{2}EI\left(\cos\beta l-cos\;\delta l\right)}{2\sqrt{\Delta }} & +\frac{\beta \delta EI\left(\delta \sin\beta l-\beta \sin\delta l\right)}{2\sqrt{\Delta }} & \frac{\beta^{2}cos\beta l-\delta^{2}\;cos\;\delta l}{2\sqrt{\Delta }} & -\frac{\delta \sin\delta l-\beta \sin\beta l\;}{2\sqrt{\Delta }}\\ \frac{\beta^{2}\delta^{2}EI\left(\beta \;sin\;\beta l-\delta \;sin\;\delta l\right)}{2\sqrt{\Delta }} & +\frac{\beta^{2}\delta^{2}EI\left(\cos\beta l-cos\;\delta l\right)}{2\sqrt{\Delta }} & -\frac{{\beta^{3}\;sin\;\beta l-\delta }^{3}\;sin\;\delta l}{2\sqrt{\Delta }} & \frac{\beta^{2}\;cos\;\beta l-\delta^{2}\;cos\;\delta l}{2\sqrt{\Delta }} \end{array}\right]\left\{\begin{array}{c} v_{0}^{\prime }\\ \varphi_{0}^{\prime }\\ M_{0}^{\prime }\\ T_{0}^{\prime } \end{array}\right\},$$

## 3. Results

### 3.1. Results for a Double Articulated Buckling Bar in a Rigid Environment

With the conditions (39) the matrix relation (38) becomes (75):

(75) $$\left\{\begin{array}{c} 0\\ \varphi \left(l\right)\\ 0\\ T\left(l\right) \end{array}\right\}=\left[\begin{array}{cccc} 1 & l & \frac{1-\cos\beta l}{EI\beta^{2}} & \frac{\beta l-sin\beta l}{EI\beta^{3}}\\ 0 & 1 & -\frac{sin\beta l}{EI\beta } & -\frac{cosl\beta 1}{EI\beta^{2}}\\ 0 & 0 & \frac{cos\beta l}{EI} & +\frac{sin\beta l}{EI\beta }\\ 0 & 0 & +\frac{\beta sin\beta l}{EI} & -\frac{\cos\beta l}{EI} \end{array}\right]\left\{\begin{array}{c} 0\\ \varphi_{0}\\ 0\\ T_{0} \end{array}\right\},$$

The trivial solution (76) is removed:

(76) sinβl=0,

The first accepted solution is (77):

(77) βl=π,

and (78):

(78) $$\beta =\frac{\pi }{l},$$

or, (79):

(79) $$\beta^{2}=\frac{\pi^{2}}{l^{2}},$$

With (79) and notation (7), relation (80) can be written as:

(80) $$\beta^{2}=\frac{\pi^{2}}{l^{2}}=\frac{P}{EI},$$

and the critical buckling force is given by (81):

(81) $$P_{cb}=\frac{\pi^{2}EI}{l^{2}},$$

That is Euler’s formula, a value identical to that calculated in Ref. [2].

### 3.2. Results for a Dental Implant as a Double-Articulated Buckling Bar in an Elastic Environment

The elastic medium can be perfectly uniform and homogeneous. The function for the elasticity coefficient of the elastic environment is denoted as *k*(*x*). It is assumed, as a working hypothesis, that the deflections at the two ends of the bar are initially zero.

The conditions at the two ends of the doubly articulated bar can be expressed as (82):

(82) $$\left\{\begin{array}{c} v_{0}^{\prime }=0\\ M_{0}^{\prime }=0\\ v\prime \left(l\right)=0\;\\ M\prime \left(l\right)=0 \end{array}\right.,$$

With (82) the matrix relation (74) becomes (83):

(83) $$\left\{\begin{array}{c} 0\\ \varphi \prime \left(l\right)\\ 0\\ T\prime \left(l\right) \end{array}\right\}=\left[\begin{array}{cccc} \frac{\beta^{2}cos\delta l-\delta^{2}cos\beta l}{2\sqrt{\Delta }} & -\frac{\delta^{3}\sin\beta l-\beta^{3}\sin\delta l}{2\beta \delta \sqrt{\Delta }} & -\frac{cos\beta l-cos\delta l}{2EI\sqrt{\Delta }} & -\frac{\mathrm{\delta sin}\beta l-\beta \sin\delta l}{2\beta \delta EI\sqrt{\Delta }}\\ \frac{\beta \delta \left(\delta sin\beta l+\beta sin\delta l\right)}{2\sqrt{\Delta }} & \frac{{\beta^{2}\cos\delta l-\delta }^{2}\cos\beta l}{2\sqrt{\Delta }} & +\frac{\beta sin\beta l-\delta sin\delta l}{2EI\sqrt{\Delta }} & -\frac{cos\beta l-cos\delta l}{2EI\sqrt{\Delta }}\\ +\frac{\beta^{2}\delta^{2}EI\left(\cos\beta l-cos\delta l\right)}{2\sqrt{\Delta }} & +\frac{\beta \delta EI\left(\delta \sin\beta l-\beta \sin\delta l\right)}{2\sqrt{\Delta }} & \frac{\beta^{2}cos\beta l-\delta^{2}cos\delta l}{2\sqrt{\Delta }} & -\frac{\delta \sin\delta l-\beta \sin\beta l}{2\sqrt{\Delta }}\\ \frac{\beta^{2}\delta^{2}EI\left(\beta sin\beta l-\delta sin\delta l\right)}{2\sqrt{\Delta }} & +\frac{\beta^{2}\delta^{2}EI\left(\cos\beta l-cos\delta l\right)}{2\sqrt{\Delta }} & -\frac{{\beta^{3}sin\beta l-\delta }^{3}sin\delta l}{2\sqrt{\Delta }} & \frac{\beta^{2}cos\beta l-\delta^{2}cos\delta l}{2\sqrt{\Delta }} \end{array}\right]\left\{\begin{array}{c} 0\\ \varphi_{0}^{\prime }\\ 0\\ T_{0}^{\prime } \end{array}\right\},$$

or, (84):

(84) $$\left\{\begin{array}{c} 0\\ \varphi \prime \left(l\right)\\ 0\\ T\prime \left(l\right) \end{array}\right\}=\left[\begin{array}{cccc} a_{11} & a_{12} & a_{13} & a_{14}\\ a_{21} & a_{22} & a_{23} & a_{24}\\ a_{31} & a_{32} & a_{33} & a_{34}\\ a_{41} & a_{42} & a_{43} & a_{44} \end{array}\right]\left\{\begin{array}{c} 0\\ \varphi_{0}^{\prime }\\ 0\\ T_{0}^{\prime } \end{array}\right\},$$

with the following notations for each line:

- for the first line as shown in (85):

(85) $$\left\{\begin{array}{c} a_{11}=\frac{\beta^{2}cos\;\delta l-\delta^{2}cos\;\beta l}{2\sqrt{\Delta }}\\ a_{12}=-\frac{\delta^{3}\sin\beta l-\beta^{3}\sin\delta l}{2\beta \delta \sqrt{\Delta }}\\ a_{13}=-\frac{cos\;\beta l-cos\;\delta l}{2EI\sqrt{\Delta }}\\ a_{14}=-\frac{\delta \sin\beta l-\beta \sin\delta l}{2\beta \delta EI\sqrt{\Delta }} \end{array}\right.,$$

- for the second line as shown in (86):

(86) $$\left\{\begin{array}{c} a_{21}=\frac{\beta \delta \left(\delta \;sin\;\beta l+\beta \;sin\;\delta l\right)}{2\sqrt{\Delta }}\\ a_{22}=\frac{{\beta^{2}\cos\delta l-\delta }^{2}\cos\beta l}{2\sqrt{\Delta }}\\ a_{23}=+\frac{\beta sin\;\beta l-\delta \;sin\;\delta l}{2EI\sqrt{\Delta }}\\ a_{24}=-\frac{cos\;\beta l-cos\;\delta l}{2EI\sqrt{\Delta }} \end{array}\right.,$$

- for the third line as shown in (87):

(87) $$\left\{\begin{array}{c} a_{31}=+\frac{\beta^{2}\delta^{2}EI\left(\cos\beta l-cos\;\delta l\right)}{2\sqrt{\Delta }}\\ a_{32}=+\frac{\beta \delta EI\left(\delta \sin\beta l-\beta \sin\delta l\right)}{2\sqrt{\Delta }}\\ a_{33}=\frac{\beta^{2}cos\beta l-\delta^{2}\;cos\;\delta l}{2\sqrt{\Delta }}\\ a_{34}=-\frac{\delta \sin\delta l-\beta \sin\beta l\;}{2\sqrt{\Delta }} \end{array}\right.,$$

- for the fourth line as shown in (88):

(88) $$\left\{\begin{array}{c} a_{41}=\frac{\beta^{2}\delta^{2}EI\left(\beta \;sin\;\beta l-\delta \;sin\;\delta l\right)}{2\sqrt{\Delta }}\\ a_{42}=+\frac{\beta^{2}\delta^{2}EI\left(\cos\beta l-cos\;\delta l\right)}{2\sqrt{\Delta }}\\ a_{43}=+\frac{\beta^{2}\delta^{2}EI\left(\cos\beta l-cos\;\delta l\right)}{2\sqrt{\Delta }}\\ a_{44}=+\frac{\beta^{2}\delta^{2}EI\left(\cos\beta l-cos\;\delta l\right)}{2\sqrt{\Delta }} \end{array}\right.$$

The matrix relations (83) and (84) can be written as a linear system of four equations with four unknowns, which can be solved relatively easily, especially if software is designed to solve problems using the TMM. The solutions allow the determination of the critical buckling force for a bar articulated at both ends, with the lower end on an elastic medium. The bar is subjected to compression by a vertical axial force—like a simplified model of a dental implant—and the elastic medium is assimilated as the bone. The bone is analogous to an elastic medium. These results are purely theoretical, our study being theoretical for now. It is a building block for our future studies which will be carried out on models as close to reality as possible. The results we obtained through the TMM will be validated through FEM and experimental determinations.

## 4. Discussion

This study was carried out in two steps. Initially, the bar was assumed to be resting on a rigid environment. The conditions at the two end supports were defined, and the critical buckling force was determined as in [2,3], which is very important for selecting the most suitable material for the respective dental implant [11,13,30]. The second step involved studying the same doubly articulated bar subjected to compression by a concentrated vertical axial force. In this case, it was assumed that the joint at the lower end of the bar is situated on an elastic medium. The bone in which the implant is placed is considered an elastic environment [29,31,35]. In both cases, the modeling was performed using the TMM [1,26].

In the first case, for the bar on a rigid environment, the analysis started from the differential equation of the deformed neutral fiber as in [2]. The differential equation was derived twice, resulting in a characteristic equation, whose solution gives the expression for the deflection. This expression was then differentiated four times to obtain expressions for the rotation, bending moment, and shear force at a section of the doubly articulated bar subjected to a concentrated vertical axial compression force. For this case, where the bar is on a rigid environment, the four expressions (for deflection, rotation, bending moment, and shear force) were transcribed into a matrix relation, written for specific section x [1]. The four elements (deflection, rotation, bending moment, and shear force) form a state vector associated with section x. This state vector is obtained by multiplying the transfer matrix corresponding to section x with the state vector of origin, section 0.

Then, in this matrix relation, *x* = *l* was applied. The result was the state vector of the last section, the top section of the bar, which depends on the transfer matrix and the state vector of the origin, Ssection 0. In this final matrix relation, the conditions at the two articulated supports at the ends of the bar can be applied. The matrix relation can then be developed into a linear system of four equations with four unknowns, the solution of which provides the unknown elements of the two state vectors: the state vector of the first section (section 0) and the state vector of the last section. Subsequently, the critical buckling force can be determined, a value that can also be verified using classical calculations from the Theory of Structures [2,3].

The second case involves the scenario where the dental implant is assimilated, that is, a doubly articulated bar. The bar has its lower joint situated on an elastic medium, with the mandibular bone being treated as an elastic environment. This association is an original concept, which led to this study [5]. In this case, a term involving the elasticity of the medium is introduced in the differential equation of the deformed neutral fiber. The differential equation is then derived twice, resulting in a solution for the deflection at a section x. Next, the expression for the deflection is differentiated three times to obtain the expressions for rotation, bending moment, and shear force. The four expressions (for deflection, rotation, bending moment, and shear force) were transcribed into a matrix relation, just as in the case of the buckling bar on a rigid environment, for a section *x*. For *x* = *l*, the state vector of the last section, the top section of the bar, is obtained. This state vector depends on the transfer matrix and the state vector of the origin section 0.

Now, it is possible to apply the conditions at the two ends. The matrix relation was developed into a linear system of four equations with four unknowns. The solution of this system provides the remaining two unknown elements of the state vector for the origin section and the two unknown elements of the state vector corresponding to the last section of the buckling bar on an elastic medium. After that, it is possible to calculate the state vector at any section of the bar, which contains the four elements.

Being a very advantageous method, the TMM offers the possibility to program the presented calculation algorithms, thus providing quick solutions in exceptional situations in orthodontics [34,36].

This research is currently purely theoretical and represents a first step in a new approach to the dental implant, viewed as a bar subjected to buckling, with the bone modeled as an elastic medium [31,35]. Next, we aim to develop software based on the mathematical algorithm presented in this article, which we plan to integrate with a shape optimization program. Once we have functional software based on TMM modeling, we will be able to address the issue of materials as well, since we will be able to incorporate their mechanical properties and loading conditions into the program, as close as possible to real-life scenarios [11,13,30]. We will also be able to incorporate the characteristics of the bone (such as the degree of porosity), which will influence the results, with the shape optimization program also coming into play [17,23,29]. At that stage, it will be possible to compare the results obtained for different materials and, implicitly, the associated costs. The price, like the material itself, is an important factor in the selection of the implant—a decision that will be made jointly by the doctor and the patient. Additionally, the numerical results obtained through TMM modeling can be validated against those obtained through FEM modeling, [28]. The advantage of TMM modeling and of a calculation code based on this algorithm lies in the ability to run the program as many times as needed, with results generated instantly. These results can then be discussed on the spot by the dentist with the patient involved. In this way, the best decisions can be made with full awareness and understanding, both by the doctor and the patient. The constraints can primarily be set by the doctor, following the specialist consultation and assessment of the patient’s condition [5,14,16]. However, the patient is also directly involved and can express personal preferences (such as preferred material, cost considerations, etc.). All of this can take place within the specialist’s office, and the decisions can be made immediately by those involved. We plan to carry out experimental tests in the laboratory—using testing machines—on various dental implants made from different materials currently used in implantology. Our goal is to validate the experimental results against those obtained from the two types of modeling—the TMM and the FEM—which are equivalent numerical methods. We are at the first step of our journey, but we hope to complete all the following steps, which we have briefly outlined here. In this way, we aim to contribute to the simplification of the process of selecting and designing a dental implant through the dissemination of our research results, starting with this initial step presented in the current article, [4].

## 5. Conclusions

The exploratory nature of our study is purely theoretical, with our main objectives being the application of the TMM for the study of a bar articulated at both ends, loaded to buckling in two cases: the bar on a rigid medium and the bar with the lower joint on an elastic medium. Both cases were analyzed with the TMM. In the first case, we obtained Euler’s formula, known from the Strength of Materials. In the second case, we followed the same reasoning as in the first case but considering the elastic medium. In this second case, we obtained the theoretical calculation formulas with the TMM too.

The simplified application model of this approach in the second case allowed us to think about a possible application of our study, first to a simplified model of a dental implant.

The TMM is an easy and elegant method for solving different problems in various fields, especially where iterative calculus is needed and where results must be obtained quickly. The TMM is a promising and effective method to analyze, for example, implant mechanics.

This work presents an original idea: to assimilate the dental implant as a buckling bar articulated at both ends on an elastic medium, with the mandibular bone treated as an elastic medium. The buckling bar is analyzed using the TMM, the problem that was theoretically solved by this work.

The TMM is faster than the FEM because calculus is between matrices and vectors, it is very easy to program and the results are instantaneous. The TMM can be used in iterative problems, where it is useful to quickly run several variants in order to choose the optimal solution.

This work will contribute to future research on dental implants. The TMM is very easy to program and can be used for theoretical tests aimed at optimizing implant shapes, and later, to validate theoretical results with the FEM and experimental tests both in the laboratory and in situ.

The application related to the dental implant was an inspiration for us, a challenge. The bar loaded in buckling on an elastic medium by the TMM was a challenge. We were happy that we managed to complete the analytical calculations. We hope that this first step will be a good omen for our future research.

## Data Availability

This is not the case.

## Abbreviations

The following abbreviations are used in this manuscript:

TMM	Transfer-Matrix Method
Ref.	Reference
Refs.	References
FEM	Finite Element Method
